# An alternative Semi-Autotropic Hydroponics (SAH) substrate for cassava rapid propagation: A first study case

**DOI:** 10.1371/journal.pone.0311437

**Published:** 2024-12-26

**Authors:** Mamy Makumbu Binzunga, Kintche Kokou, Sikirou Mouritala, Adetoro Najimu, Dieng Ibnou, Kajibwami Angelique, Jacob Mignouna, Aggrey Bernard Nyende

**Affiliations:** 1 International Institute of Tropical Agriculture, Bukavu-Kalambo, Democratic Republic of the Congo; 2 Institut National pour l’Etude et la Recherche Agronomiques, Kinshasa, Democratic Republic of the Congo; 3 Jomo Kenyatta University of Agriculture and Technology, Nairobi, Kenya; 4 International Institute of Tropical Agriculture, Kinshasa, Democratic Republic of the Congo; 5 International Institute of Tropical Agriculture, Ibadan, Nigeria; United States Department of Agriculture, UNITED STATES OF AMERICA

## Abstract

The expansion of Semi-Autotrophic Hydroponics technology to address the issue of multiplying and disseminating virus-free planting materials for vegetatively propagated crops is challenged by the utilization of imported substrate, namely, KlasmannTS3. In this study, we evaluated the growth parameters and cutting production of cassava genotypes during three subsequent plantlet production cycles using three single substrates, namely, KlasmannTS3 (K), vermiculite (V), and local peat (P), and three blended substrates. The blended substrates were a combination of 25% K and 75% P (K_25_P_75_), a combination of V and P at respective rates of 25% and 75% (V_25_P_75_), and respective rates of 10% and 90% (V_10_P_90_). All cuttings obtained in one plantlet production cycle were transplanted into the next. The multiplication rate of cutting from cycle 1 to 2 (R1) and cycle 2 to 3 (R2) was calculated as the ratios of the number of cuttings per the number of plantlets in each cycle. K and K_25_P_75_ led to similar R1 and R2, except with the genotype IBA961089A, where K_25_P_75_ led to a higher R1. Local peat and V solely showed similar cutting multiplication rates, and were lower than V_25_P_75_ and V_10_P_90_. Substrates with a higher cutting production also led to a higher plantlet height, leaf, and internode number. V and its combinations with local peat led to the densest plantlet root system. The performance of the substrates contrasted among the genotypes, but IBA961089A mostly outperformed the two other genotypes. We concluded that up to 75% of K and, to a lesser extent 75% of V, can be substituted by P without compromising cutting production. V and P should be combined instead of being used separately.

## Introduction

Cassava (*Manihot esculenta*) is among the most important crops in sub-Saharan Africa, particularly in the Democratic Republic of Congo (DR Congo). It plays a crucial role of ensuring food security, generating income for smallholder farmers, and supporting industrial development [1–3]. The adaptability of cassava in diverse agro- ecological conditions and its tolerance of soil fertility depletion enhance the crop’s resilience and sustainability in the context of climate change [4–6]. The DR Congo is the second-largest cassava producer in sub-Saharan Africa, following Nigeria, with total production that exceeded 40 million tons of cassava root in 2021 [7]. Cassava is a primary source of food and a cash crop for more than 70% of the country’s population [4, 5].

Despite its significance, cassava faces challenges in rapid propagation due to its low multiplication rate and reliance on vegetative propagation, which involves using stem cuttings [4–6, 8].

The conventional method of cassava propagation is inefficient, as planting materials harvested from one hectare can plant less than seven hectares after one year. This low propagation rate hinders the timely distribution of improved cassava varieties and delays material disseminating across countries due to strict regulations requiring virus-free certification, which can take up to four years [9–12].

In addition, at certain stages in the breeding scheme, high and homogeneous amounts of cassava planting materials are required for the multi-location trials [6, 7]. Moreover, although vegetative propagation preserves the crop’s desirable traits, it presents challenges related to viral infections, such as Cassava Mosaic Disease (CMD) and Cassava Brown Streak Disease (CBSD), which can cause yield losses of up to 70–100% in susceptible varieties [13–15]. These diseases result in annual production losses exceeding one billion USD and pose a threat to food security for millions of farmers in East and Central Africa [11, 12].

The spread of viruses through the utilization of infected materials is increasing as farmers plant new fields by collecting materials from their old fields or neighboring farms due to a shortage of planting materials [13, 14]. To address these challenges, smart technologies like Semi-Autotrophic Hydroponics (SAH) have been adopted by the International Institute of Tropical Agriculture (IITA) for rapid propagation of cassava and subsequently for yam (*Dioscorea spp*) in 2016 after several drawbacks of different propagation technologies [15]. SAH utilizes portions of vitro plants in laboratories and recently, small cuttings in greenhouses, to produce, in a short time, massive virus-free planting materials [16]. It is readily utilized for commercial seed production, and enhances multiplication rate in breeding programs. Recently, SAH laboratories have been established in other African countries like DR Congo, Malawi, Tanzania, and Zambia [9, 15–20]. The technology is now being adapted to sweet potato (*Solanum tuberosum*) and other crops at IITA Kalambo in DR Congo [21]. SAH benefits include a high propagation ratio of planting material in laboratory and the ability to propagate true-to-type [22–25], virus-free planting materials in reduced space and time and improved crop performance setting it apart from other technologies [26–30]. The unique substrate used under SAH technique for planting material production refers to the KlasmannTS3, which is globally renowned for its properties that enhance plant performance [30]. Studies in Nigeria and DR Congo have demonstrated better laboratory survival rates and improved cassava growth with KlasmannTS3 [31, 32]. Similar results have been also reported on yam (*Dioscorea spp*) and pineapple (*Ananas comosus*) crops [33, 34].

The expansion of SAH technique in DR Congo holds great promise for improving cassava production and food security. However, the reliance on the imported KlasmannTS3, essential for SAH, poses a significant cost barrier. To promote wider adoption of SAH, exploring alternative substrates that are cost-effective and meet plant requirements is crucial [23–25]. It has been pointed out that a convenient substrate should not only supply the physical, chemical, and biological properties required by the plants but also be available, affordable, and sustainable for practical plant production [24, 35–38].

Preliminary studies in DR Congo have evaluated the use of single substrates like vermiculite and local peat, revealing acceptable survival rates and growth performance, though lower than KlasmannTS3 [26–28]. Combining KlasmannTS3 with local substrates may enhance production efficiency and reduce costs, as mixing substrates can improve physical, chemical, and biological properties required for plant growth [29–31].

On the other hand, a mix of hydroponic substrates has demonstrated numerous advantages in enhancing plant growth performance [39, 40]. Manios et al. [41] tested rice husk biochar (RB) alone or with perlite (PL) to enhance leafy vegetable growth. The combination of PL and RB as an hydroponic substrate doubled the vegetable yield compared to PL alone. Maślanka et al. [40] declared that the vermiculite structure is not very stable because of low compression resistance and tends to deteriorate over time, reducing water drainage. The authors argued that such substrate can be used alone; however, it is preferable to mix it with other substrates such as peat.

This study aimed to evaluate the growth parameters and multiplication rate of planting materials of cassava genotypes using single and combined substrates under the SAH system.

## Materials and methods

### Study location

The experiment was conducted at the SAH laboratory of the Olusegun Obasanjo Research Campus, International Institute of Tropical Agriculture (IITA), in Kalambo, South Kivu province, DR Congo (S 2°23’50", E 28°50’42", and 1,488 masl). The experiment was carried out from November 2021 to January 2022. The laboratory is equipped with standard materials and is being fully operated to produce plantlets of many crops, including cassava.

### Experimental design

The experiment was laid out in a split-plot design with four replicates, with cassava genotype as the main factor and substrates as the sub factor.

### Source and description of study materials

Three improved genotypes were used in this study, comprising introduced clones (IBA961089A, IBA70520, and IBA980505) under evaluation at the IITA Kalambo station. The genotypes were selected for their fast recovery from cutting in the laboratory, fast growth, wide adaptability in the field, and high-yielding traits. All genotypes used were resistant to cassava mosaic disease and had a straighter growth habit. They originated from four-week-old mother plantlets derived from tissue culture.

### Substrate preparation

Three single substrates and three blended substrates were tested. The single substrates are KlasmannTS3, vermiculite, and locally sourced peat. KlasmannTS3 primarily consists of white and black peat, supplemented with organic and mineral materials such as wood fiber, green compost, and coconut fiber [18]. Vermiculite has a medium particle size. The local peat is an organic matter collected from a farm in Bukavu town, in an undeveloped area that is temporarily flooded and covered with a thin layer of vegetation for long period (S 2°40’42", E 28°46’58", 1,934 m). Treatment of local peat involved sterilization at 121°C for 15 minutes, followed by a cooling period of 24 hours [39]. The blended substrates are the combination of (i) KlasmannTS3 and local peat at respective rates of 25% and 75% (K_25_P_75_), (ii) vermiculite and local peat at respective rates of 10% and 90% (V_10_P_90_), and (iii) vermiculite and local peat at the respective rate of 25% and 75% (V_25_P_75_). The fraction of the substrate in the combinations refers to the volume. For each substrate, 500 ml of volume was put into a transparent light. For example, in the K_25_P_75_ combination, 125 ml of KlasmannTS3 and 375 ml of local peat were used. Due to differences in bulk density, the weight of KlasmannTS3 and local peat would not correspond exactly to 25% and 75%, respectively.

### Subculture cutting production

The experiment consisted of growing plantlets of a genotype in the same substrate for three successive cycles of plantlet production. Each production cycle had a four-week duration. As starting materials, mother plantlets (4 weeks old) obtained from tissue culture, were used for each genotype. The mother plantlets were produced once in a common substrate (KlasmannTS3). During the first production cycle, 500 ml of each substrate were put into the transparent light box of 15 cm × 15 cm × 9 cm, in which twenty cuttings of the mother plants were planted in a regular space of 3 cm × 3 cm. Each planted cutting had a length of at least 1 cm and contained one node and one not fully developed leaf. The plantation consisted of inserting 0.5 cm of the cutting portion into the substrate. The second production cycle was established by transplanting all cuttings, which the plantlets of a genotype grew in a specific substrate in cycle 1 were able to produce at the end of four weeks. When, for a genotype × substrate, the cuttings obtained from cycle 1 were more than 20 (i.e., cutting number to be transplanted in a box with space of 3 cm × 3 cm), boxes of the concerned substrate were added. When for a genotype × substrate treatment, the obtained cuttings were less than 20 or while the number of remaining cuttings to be transplanted in a new box was less than 20, the cuttings were transplanted in the same space of 3 cm × 3 cm, and then a portion of substrate in the box was left empty. The third cycle of plantlets was established in the same way using all cuttings, that plantlets of a genotype grew in a substrate in cycle 2 were able to produce at the end of four weeks.

The 500 ml of substrate per box was watered with 100 ml of Miracle-Gro All-Purpose Water Solution (2.6 gl/4L) as the nutrient source, at the transplanting time and after that once a week during the four weeks of cycle duration. Plantlets were grown in a controlled environment with a temperature of 25 ± 20°C, a light intensity of 20 w [30, 31], and a photoperiod of 10 hours of light and 14 hours of darkness per day. The SAH box lids were kept closed during the growth period to reduce transpiration.

### Substrate analysis

The Kjeldahl digestion method [42] was employed to quantify the total nitrogen (N) content of the substrates. Substrate pH and electrical conductivity (EC) were determined using the electrometric method [43]. For the analysis of exchangeable cations, including calcium (Ca^2^⁺), magnesium (Mg^2^⁺), and potassium (K⁺), along with cation exchange capacity (CEC), the ammonium acetate extraction method was utilized [44, 45]. Available phosphorus (P) was determined using the Bray 1 method [46].

### Data collection

The number of cuttings produced by cassava plantlets of a given genotype, growing in a substrate, was manually counted and recorded at four weeks of age during each production cycle. In each plantlet production cycle, cuttings that sprouted were counted from the second week after transplanting (WAT), with weekly counts continuing until 4 WAT for each cassava genotype and per substrate. In each plantlet production cycle, researchers counted the cuttings that sprouted starting from the second week after transplanting (WAT), with weekly counts continuing until 4 WAT for each cassava genotype and per substrate. Data including height (cm) of plantlets, number of leaves and internodes per plantlet, were collected at the same growth stages in each plantlet production cycle from five plantlets randomly selected. Height was measured from the base to the newly emerging leaf of the plantlets using a measuring tape, while leaves and internodes were counted manually on all selected plantlets. Leaf absorbance light capacity was measured (SPAD Units), which is proportional to the amount of chlorophyll present in the leaves of five randomly selected plantlets per genotype and substrate using the SPAD-502 Plus device (Konica Minolta, Inc.). Three leaves per plantlet were assessed and the average absorbance capacity per plantlet was recorded. The SPAD-502 Plus is a hand-held device used to determine leaf absorbance capacity by inserting plant leaf into the receptor window and then closing the measuring head with fingers [47]. At the end of each cycle, we uprooted plantlets of one replication: (i) to count the main and secondary roots and (ii) to measure the length of the main root in the five randomly selected plantlets per genotype and substrate.

### Data analysis

The multiplication rate of transplanted material from one cycle to the next was assessed using the following ratios:

$$R1=\frac{Number\;of\;cuttings\;obtained\;in\;cycle\;1\;and\;transplanted\;into\;cycle\;2}{Number\;of\;plantlets\;alive\;at\;the\;end\;of\;cycle\;1}$$ (1)

$$R2=\frac{Number\;of\;cuttings\;obtained\;in\;cycle\;2\;and\;transplanted\;into\;cycle\;3}{Number\;of\;plantlets\;alive\;at\;the\;end\;of\;cycle\;2}$$ (2)

R1 or R2 less than 1 indicates that at the end of the cycle, some plantlets were unable to produce a single cutting for transplanting into the next cycle. R1 or R2 greater than 1 indicates that at least one plantlet produced at least two cuttings by the end of the cycle. R1 and R2 were calculated for each cassava genotype and each substrate.

The survival rate of cuttings/plantlets was calculated as the percentage of plantlets surviving at the observation period compared to the initial number of cuttings transplanted:

$$Survival\;rate\;(\%)=\frac{Number\;of\;surviving\;cuttings\;or\;plantlets\;}{Number\;of\;cuttings\;transplanted}\;X\;100$$ (3)

The survival rate was calculated for each substrate and per genotype at the end of the 2nd, 3rd, and 4th week in cycles 1, 2, and 3 of plantlet production.

Data were analyzed for each cycle using a linear mixed model accounting for correlations between responses measured over three weeks on the same units. A repeated measurement setting was used, assuming temporal correlations. Genotype, substrate, and week along with their 2-way and 3-way interactions, were considered as fixed effects, while replicate and genotype × replicate were random effects. Leaf absorbance capacity and plantlet height were analyzed under a normal distribution. A generalized mixed model [48] was used for leaf and internode numbers (Poisson distribution) and survival rate (binomial distribution).

We tested for a significant genotype × substrate × week interaction effect. If a significant interaction was found, we then fitted a model for each week separately. Predicted means were computed and Tukey’s honest significant difference (Tiukey’s HSD) test was used for comparison and ranking. Analyses were conducted using the MIXED and GLIMMIX procedures in SAS/STAT software, Version 9.4 for Windows [36].

## Results

### Chemical and physical characteristics of the substrates

The local peat and its combinations with KlasmannTS3 or vermiculite were more acidic than single vermiculite and KlasmannTS3 (Table 1).

**Table 1:** Chemical characteristics and nutrient concentration of the substrates used to produce cassava plantlets under the SAH system.

Substrate	pH (H_2_O)	Weight of 500 ml (g)	Total N (g kg^-1^)	Exch. K (g kg^-1^)	Exch. Ca (g kg^-1^)	Exch. Mg (g kg^-1^)	Av. P (g kg^-1^)	CEC (cmol kg^_1^)	EC (μS cm^_1^)	NS (mlg^_1^) of substrate
**KlasmannTS3**	5.86	135	7.8 (1.1)	1.2 (0.2)	51.7 (7.0)	2.9 (0.4)	0.8 (0.1)	57.8	247.1	0.74
**Local peat**	3.74	205	13.8 (2.8)	2.4 (0.5)	20.9 (4.3)	0.0 (0.0)	0.6 (0.1)	71.9	91.4	0.49
**Vermiculite**	5.23	200	0.5 (0.1)	20.4 (4.1)	46.6 (9.3)	124.8 (25.0)	0.4 (0.1)	6.3	8.6	0.5
**V**_**10**_ **P**_**90**_	3.69	204	12.5 (2.6)	4.2 (0.9)	23.5 (4.8)	12.5 (2.6)	0.6 (0.1)	65.4	83.1	0.49
**K**_**25**_ **P**_**75**_	3.74	187	12.3 (2.3)	2.1 (0.4)	28.6 (5.4)	0.7 (0.1)	0.7 (0.1)	68.4	130.3	0.53
**V**_**25**_ **P**_**75**_	4.07	204	10.5 (2.1)	6.9 (1.4)	27.3 (5.6)	31.2 (6.4)	0.6 (0.1)	55.5	70.7	0.49

Value in brackets corresponds to the total nutrient amount (g) in 500 ml of substrate used per box to produce the plantlets. They were calculated using substrate weight (2^nd^ column) and the corresponding nutrient concentration. Exch: exchange, Av: available, pH: hydrogen potential, N: nitrogen, K: potassium, Ca: calcium, Mg: magnesium, P: phosphorous, CEC: cation exchange capacity, EC: electrical conductivity, NS: nutrient solution periodically added during plantlet’s growth.

The pH of local peat and its combinations with Klasmann TS3 or vermiculite ranged from 3.7 to 4.1, while single vermiculite and Klasmann TS3 had pH values of 5.2 and 5.9, respectively. For the same volume (500 ml), local peat and vermiculite weighed 205 g and 200 g, respectively, both higher than Klasmann TS3 at 135 g. Local peat had an average N content of 13.8 g/kg, 1.8 times higher than Klasmann TS3 (7.8 g/kg). Due to its higher weight and N content, the N amount per box of local peat was 2.7 times that of KlasmannTS3. Vermiculite had a significantly lower N content (Table 1).

The blended substrates (V_10_P_90_, V_25_P_75_, and K_25_P_75_) had higher N content compared to single KlasmannTS3 and vermiculite due to the high N content and weight of local peat. Vermiculite and local peat had exchangeable K values of 20.4 g/kg and 2.4 g/kg, respectively, which were 17 and 2 times higher than KlasmannTS3 (1.2 g/kg). Consequently, blended substrates had a higher exchangeable K content than single KlasmannTS3.

Exchangeable Mg in vermiculite averaged 125 g/kg, significantly higher than in other substrates (Table 1). Blended substrates with 10% or 25% vermiculite had higher exchangeable Mg content due to vermiculite’s high Mg levels. Substituting 25% of local peat with KlasmannTS3 resulted in a lower exchangeable Mg content.

KlasmannTS3 had the highest exchangeable Ca content (52 g/kg). However, due to its lower weight, the total Ca amount per box (7 g) was slightly lower than vermiculite (9 g). Local peat and blended substrates had slightly lower total Ca amounts per box than KlasmannTS3. The P content was low and similar across all substrates. The nutritive solution applied was 0.5 ml/g for local peat, vermiculite, and blended substrates, lower than 0.7 ml/g for KlasmannTS3 (Table 1). Vermiculite had markedly low CEC and electrical conductivity compared to other substrates.

Test of the effects of genotype, substrate, and growth stage and their interactions on survival rate and agronomic performance over three successive production cycles under the SAH system

### Multiplication rate of cassava cuttings from one cycle to the next

**Fig 1:**
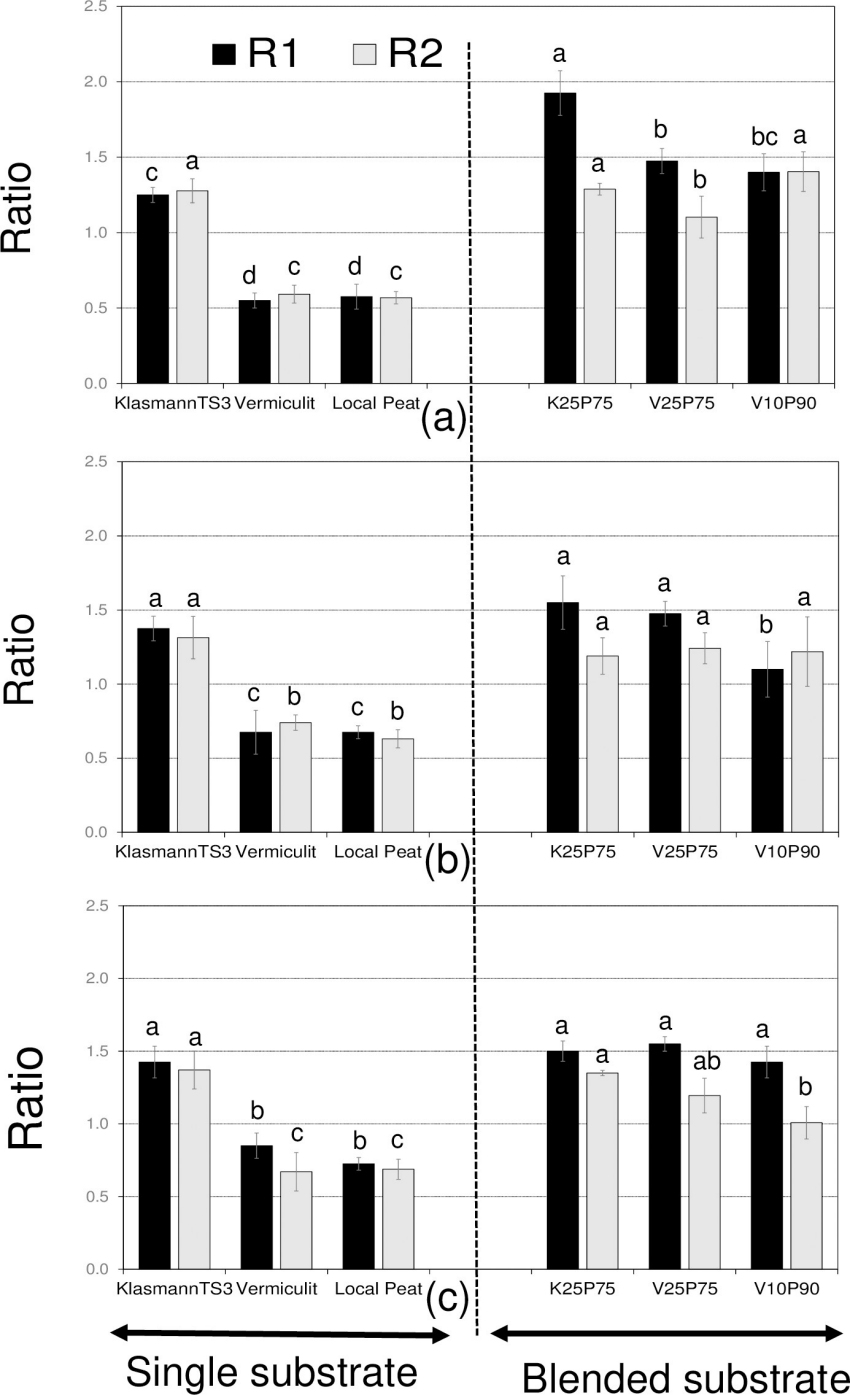
Cutting multiplication rate from cycle 1 to cycle 2 (R1) and from cycle 2 to cycle 3 (R2) of three cassava genotypes across six substrates. (a): IBA961089A, (b): IBA70520, and (c): IBA980505. R1 and R2 with different letters in the same substrate are significantly different (p< 0.05) using Turkey’s Honest Significant Difference Test.

Regardless of cassava genotype, the number of cuttings obtained and transplanted into the next cycle was higher with blended and single KlasmannTS3 substrates compared to single vermiculite and local peat (Fig 1).

Using blended and single KlasmannTS3 substrates resulted in more cuttings transplanted into the next cycle compared to single vermiculite and local peat, regardless of genotype (Fig 1). For instance, with blended local peat and KlasmannTS3 (K_25_P_75_), R1 averaged 1.92 for IBA961089A (Fig 1A). This indicates that the number of IBA961089A cuttings obtained in cycle 1 and transplanted into cycle 2 was nearly double the number of plantlets that survived in cycle 1. A similar trend was observed with combined local peat and vermiculite (V_10_P_90_ and V_25_P_75_), though their multiplication rates were not as high as K_25_P_75_, with increases of 40% and 50%, respectively. The multiplication rate from cycle 2 to cycle 3 was lower than that from cycle 1 to cycle 2.

The number of IBA961089A cuttings obtained in cycle 2 and transplanted into cycle 3 (R2) increased by 40%, 30%, and 10% for V_10_P_90_, K_25_P_75_, and V_25_P_75_, respectively. Single KlasmannTS3 led to a significantly lower (p<0.01) multiplication rate from cycle 1 to cycle 2 (30% versus 40%–92% for blended substrates) (Fig 1A). From cycle 2 to cycle 3, the increase in cuttings for IBA961089A with single KlasmannTS3 averaged 30%, not differing significantly from K_25_P_75_ and V_25_P_75_ but higher than V_10_P_90_.

The same trend was observed for the other genotypes (Fig 1B and 1C). Single KlasmannTS3 resulted in a similar increase in cuttings for IBA70520 and IBA980505 compared to K_25_P_75_ and V_25_P_75_. However, single KlasmannTS3 showed a significantly higher rate of cuttings from cycle 1 to 2 for IBA70520 and from cycle 2 to 3 for IBA980505 compared to V_10_P_90_. For all genotypes, R1 and R2 were lower than 0.6 with single vermiculite and single local peat, indicating a 40% decrease in the number of cuttings transplanted from one cycle to the next.

### Plantlet survival rate

The three-way interaction (substrate × genotype × week) and two-way (substrate × genotype) interaction were not significant for plantlet survival rate (Table 2A). However, there were significant substrate × week and early-stage genotype × week interactions. The proportion of surviving plantlets, influenced by substrates over the growth period, was mainly significant with local peat and vermiculite combinations (V_10_P_90_ and V_25_P_75_) (Table 3).

**Table 2:** Significance of genotype, substrate, and/or growth stage and their 2 and 3-order interactions for agronomic parameters during 3 plantlet production cycles in the SAH laboratory.

Parameter	Plantlet production cycle	Genotype, substrate, and/or Week and their 2-order and 3-order interactions effect							
			S	G	W	S x G	S x W	G x W	S x G x W
**Survival rate (a)**	**1**		***	ns	***	ns	**	*	ns
	**2**		***	*	***	ns	*	ns	ns
	**3**		***	*	***	ns	***	ns	ns
**Plantlet height** **(b)**	**1**		***	***	***	*	***	***	***
	**2**		***	***	***	***	***	*	***
	**3**		***	*	***	ns	***	*	ns
**Leaf number (c)**	**1**		***	*	***	*	***	***	ns
	**2**		***	ns	***	*	ns	ns	ns
	**3**		***	ns	***	ns	*	*	*
**Internode** **Number** **(d)**	**1**		***	**	***	***	***	***	*
	**2**		***	*	***	***	ns	ns	ns
	**3**		***	**	***	***	ns	*	ns
**leaf absorbance capacity (e)**	**1**		***	*	***	**	***	ns	ns
	**2**		***	***	***	***	ns	ns	ns
	**3**		**	**	***	***	ns	ns	ns
**Root number (f)**			***	*		*			
**Root length (g)**			***	***		***			

S: substrate, G: genotype, W: growth stage (week), S x G x W: interaction between the 3 factors; S x G: interaction between substrate and genotype; S x W: interaction between substrate and week; G x W: Interaction between genotype and week. Significant codes:” ns “no significant

“*”0.05

“** “0.01

“***”0.001 (α < 5%).

**Table 3:** Survival rate (%) of plantlets from cassava genotypes grown in different substrates across production cycles in the SAH laboratory.

Cycle	Growth stage	Substrate						Genotype		
		K_25_P_75_	KlasmannTS3	Local Peat	V_10_P_90_	V_25_P_75_	Vermiculite	IBA70520	IBA961089A	IBA980505
**1 (A)**	**Week 2**	95^a^	98^a^	71^c^	85^b^	87^b^	73^c^	85^a^	83^a^	86^a^
	**Week 3**	94^a^	94^a^	64^d^	81^c^	87^b^	68^d^	79^a^	82^a^	83^a^
	**Week 4**	94^a^	89^ab^	63^d^	81^c^	87^b^	67^d^	76^b^	80^ab^	83^a^
**2 (B)**	**Week 2**	95^a^	97^a^	74^c^	85^b^	88^b^	74^c^	80^b^	86^a^	83^ab^
	**Week 3**	95^a^	93^ab^	70^d^	81^c^	88^b^	67^d^			
	**Week 4**	95^a^	90^ab^	70^d^	80^c^	88^b^	66^d^			
**3 (C)**	**Week 2**	94^ab^	96^a^	76^d^	85^c^	89^bc^	78^d^	81^b^	86^a^	83^ab^
	**Week 3**	94^a^	89^ab^	70^d^	81^c^	88^b^	73^d^			
	**Week 4**	93^a^	87^b^	70^d^	80^c^	88^b^	69^d^			

Means with different letters in each row are significantly different (p< 0.05) using the Turkey’s Honest Significant Difference Test (HSDT).

The survival rate varied among genotypes over the growth period in cycle 1 (significant genotype × growth stage interaction) (Table 2A) but was consistent across growth stages in cycles 2 and 3. For cycles 2 and 3, the average for all measurement periods was presented per genotype, as the contrasts among genotypes were consistent across growth stages. Although the two substrate combinations resulted in similar survival rates at the end of week 2, the combination with more local peat (V_10_P_90_) led to a lower survival rate by the end of weeks 3 and 4 compared to the combination with less local peat (V_25_P_75_). Among single substrates, KlasmannTS3 had the highest survival rate in all cycles and at each period. Single vermiculite and local peat had similar survival rates.

The combination of KlasmannTS3 and local peat (K_25_P_75_) had a similar survival rate to single KlasmannTS3 but was significantly higher than local peat alone. Combinations of vermiculite and local peat (V_10_P_90_ and V_25_P_75_) increased survival rates in all cycles compared to single vermiculite or local peat.

The survival rate varied among genotypes over the growth period in cycle 1 (Table 2A) but was consistent across growth stages in cycles 2 and 3. For IBA70520, the survival rate at the end of week 4 in cycle 1 was significantly lower than that of IBA980505 (78% vs. 84%), with no significant differences at earlier stages. In cycles 2 and 3, the survival rate for IBA70520 was consistently lower than for IBA961089A, regardless of the growth stage (81% vs. 86%).

### Effect of substrate on aerial growth parameters of cassava plantlets

#### Plantlet height

The height of the plantlets varied significantly depending on both the substrate and the cassava genotype in all production cycles (Table 4).

**Table 4:** Height (cm) of plantlets of cassava genotypes grown in different substrates across production cycles in the SAH laboratory.

Cycle	Growth stage	Genotype	Substrate					
			K_25_P_75_	KlasmannTS3	Local Peat	V_10_P_90_	V_25_P_75_	Vermiculite
**1 (A)**	**Week 2**	**Overall**	5.3^a^	5.8^a^	2.4^c^	2.8^bc^	3.0^b^	3.3^b^
	**Week 3**	**IBA70520**	7.1^a^	6.4^a^	3.8^b^	3.5^b^	3.9^b^	3.6^b^
		**IBA961089A**	7.2^b^	9.0^a^	4.0^d^	3.7^d^	4.0^d^	5.1^c^
		**IBA980505**	6.1^a^	6.3^a^	2.8^b^	3.5^b^	3.6^b^	3.5^b^
	**Week 4**	**IBA70520**	8.0^a^	7.7^a^	4.7^b^	4.5^b^	4.8^b^	4.2^b^
		**IBA961089A**	9.2^b^	10.4^a^	5.6^d^	4.3^e^	4.7^de^	7.3^c^
		**IBA980505**	7.6^a^	8.2^a^	3.6^b^	3.9^b^	4.1^b^	4.2^b^
**2 (B)**	**Week 2**	**IBA70520**	3.3^b^	4.6_a_	2.2^c^	2.5^bc^	2.1^c^	2.3^c^
		**IBA961089A**	5.9^b^	7.3^a^	2.4^d^	2.7^cd^	3.0^cd^	3.2^c^
		**IBA980505**	3.7^b^	4.7^a^	1.4^d^	2.5^c^	2.7^c^	2.6^c^
	**Week 3**	**IBA70520**	5.1^b^	6.4^a^	2.9^c^	3.2^c^	3.1^c^	3.5^c^
		**IBA961089A**	6.9^b^	9.6^a^	3.5^d^	3.6^d^	3.9^cd^	4.6^c^
		**IBA980505**	5.1^b^	6.8^a^	2.4^c^	3.5^b^	3.5^b^	3.6^b^
	**Week 4**	**IBA70520**	7.3^b^	9.2^a^	4.0^c^	4.2^c^	4.2^c^	4.9^c^
		**IBA961089A**	8.4^b^	11.2^a^	5.7^d^	5.1^d^	5.4^d^	7.0^c^
		**IBA980505**	7.5^b^	8.8^a^	3.2^c^	4.1^bc^	4.6^b^	4.3^b^
**3 (C)**	**Week 2**	**Overall**	4.9^b^	5.8^a^	2.2^c^	2.4^c^	2.6^c^	2.7^c^
	**Week 3**	**Overall**	6.2^b^	7.4^a^	3.4^c^	3.3^c^	3.6^c^	3.8^c^
	**Week 4**	**Overall**	8.6^b^	9.5^a^	4.8^de^	4.2^e^	4.8^d^	5.6^c^

Means with different letters in each row are significantly different (α < 5%) using the HSDT.

The interaction between substrate, cassava genotype, and growth stage was significant in cycles 1 and 2 (Tables 2B and 4A). Then, for cycles 1 and 2, we ran separate linear mixed models each week. The interaction between genotype and substrate was not significant during the second week, whereas it was during weeks 3 and 4 (not shown). Here, the plantlets of the genotypes IBA980505 and IBA70520 had almost similar heights in a single KlasmannTS3 and K_25_P_75_. The plantlets of these two genotypes also had similar heights in single local peat, single vermiculite, and the combinations of the two substrates (V_10_P_90_ and V_25_P_75_). While the genotype IBA961089A showed in the same cycle and same growth stages, higher plantlets in single KlasmannTS3 compared to the K_25_P_75_ substrate, and higher plantlets in single vermiculite in comparison to single local peat, V_10_P_90_, and V_25_P_75_. Irrespective of cassava genotype in cycle 1, the plantlets grown in single KlasmannTS3 and K_25_P_75_ were taller than those grown in single local peat, single vermiculite, V_10_P_90_, and V_25_P_75_ at the end of weeks 3 and 4. A similar trend occurred among the substrates at the end of week 2 of the cycle, except that the contrasts did not depend on the cassava genotype at this growth stage.

In cycle 2, the significant interaction between substrate and cassava genotype occurred at different growth stages (Table 4B). Here, an increase in the height of the plantlets grown in a single KlasmannTS3 compared to those grown in K_25_P_75_ occurred with all genotypes (with IBA980505 and IBA70520 only in cycle 1). An increase in the height of the plantlets grown in single vermiculite compared to those grown in single local peat occurred with IBA961089A and IBA980505 (with IBA961089A alone in cycle 1). As in cycle 1, the plantlets grown in single vermiculite, single local peat or in the combinations of the two substrates were short compared to those grown in single KlasmannTS3 or K_25_P_75_. In cycle 3, the interaction between genotype and substrate was not significant (Table 2B), hence the means of the substrates were estimated. Here also, a higher height of the plantlets grown in single KlasmannTS3 compared to those in K_25_P_75_, and a higher height of the plantlets grown in single KlasmannTS3 or in K_25_P_75_ compared to those grown in single local peat, single vermiculite, V_10_P_90_ and V_25_P_75_ occurred irrespective of the growth stage (Table 4C). An increase in the height of the plantlets grown in single vermiculite compared to those grown in single local peat occurred at the end of week 4 only. For a substrate, where the interaction with genotype was significant, IBA961089A were taller than those of the two other genotypes (Table 4). Even at the growth stages or cycles with no significant interaction between substrate and the cassava genotype, plantlets of the genotype IBA961089A were the tallest (unshown data). At the end of week 2 in cycle 1, the height of the plantlets of IBA961089A averaged 4.3 cm, and they were significantly taller than the plantlets of the two other genotypes, with an average height of 3.5 cm. The same was true at each measurement period in cycle 3, where the plantlets of a genotype IBA961089A averaged 4.0, 5.3, and 7.2 cm at the end of weeks 2, 3, and 4, respectively, compared to 3.2, 4.6, and 6.2 cm for the plantlets of IBA70520 and 3.0, 4.0, and 5.4 cm for the plantlets of IBA980505 at the same growth stages.

#### Leaf number

**Fig 2:**
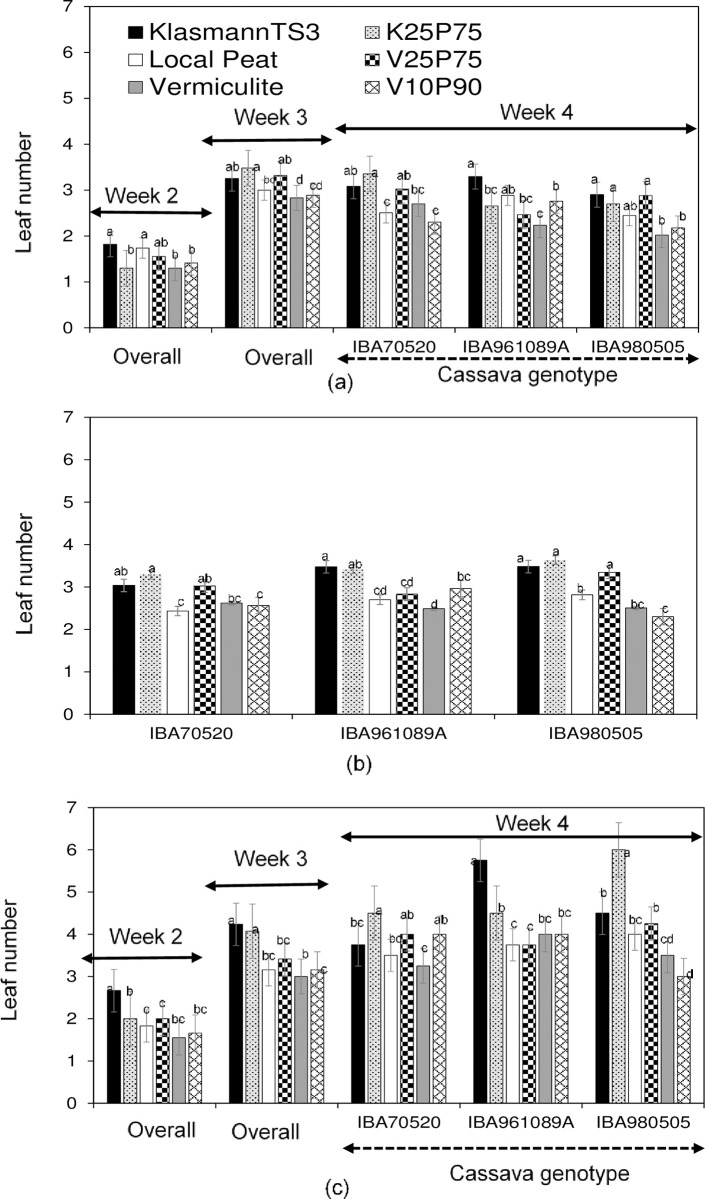
Average number of leaves (No) per plantlet at the end of weeks 2, 3, and 4 for cassava genotypes grown in different substrates across three production cycles in the SAH laboratory. (a): 1^st^ cycle, (b): 2^nd^ cycle, (c): 3^rd^ cycle. Per genotype or per week, values of the histograms with different letters are significantly different (α < 5%) using the HSDT.

Regarding the number of leaves per plantlet, the interaction between genotype, substrate, and week was significant only in cycle 3. Within cycle 3, a significant interaction between genotype and substrate was found only at week (Table 2C). In cycle 1, the interaction between substrate and the genotype was significant only at the end of the fourth week (Fig 2A).

Until the end of week 3 in both cycles 1 and 3, plantlets grown in single KlasmannTS3 developed more leaves compared to those grown in single vermiculite or local peat, regardless of the cassava genotype (Fig 2A and 2C). Compared to single KlasmannTS3, plantlets grown in the combination of KlasmannTS3 and local peat (K_25_P_75_) had fewer leaves at the end of week 2, but similar leaf numbers at the end of week 3 in both cycles. The leaf number of plantlets grown in K_25_P_75_ increased compared to single local peat by the end of week 3 in cycles 1 and 3. Plantlets grown in combinations of local peat and vermiculite (V_10_P_90_ and V_25_P_75_) had similar leaf numbers to those grown in single local peat or single vermiculite at these growth stages in cycles 1 and 3.At the end of week 4 in cycles 1 and 3, there was a significant substrate × genotype interaction, leading to different effects of the substrates among the cassava genotypes (Fig 2A and 2C). For example, in cycle 3, IBA980505 and IBA70520 plantlets had more leaves in K_25_P_75_ compared to single KlasmannTS3, whereas IBA961089A had fewer leaves in K_25_P_75_. In cycle 1, IBA961089A also had fewer leaves in K_25_P_75_ compared to the single KlasmannTS3, while the other two genotypes showed no significant difference in leaf number between these substrates. Overall, plantlets grown in single KlasmannTS3 had more leaves than those grown in single local peat or single vermiculite, with no significant difference in leaf number between plantlets grown in single local peat and single vermiculite at the end of cycles 1 and 3 (Fig 2A and 2C). In cycle 2, the significant interaction was only between substrate and genotype, with no significant interaction between growth stage and substrate or genotype (Table 2C). For instance, single KlasmannTS3, K_25_P_75_, and V_25_P_75_ resulted in similar leaf numbers for IBA980505 and IBA70520, whereas IBA961089A developed fewer leaves in V_25_P_75_ compared to K_25_P_75_ and single KlasmannTS3, regardless of the growth stage in cycle 2 (Fig 2B). Across all growth stages and genotypes in cycle 2, plantlets grown in single KlasmannTS3 and K_25_P_75_ had similar, and consistently higher, leaf numbers compared to those grown in single local peat or single vermiculite.

#### Internode number

This result indicates that while a substrate may lead to a high internode number in some cassava genotypes, it can result in a low internode number in others, demonstrating a contrasting substrate effect among the genotypes.

In cycles 2 and 3, the contrasting substrate effects on the number of internodes among cassava genotypes remained consistent throughout the plantlet growth stages. In cycle 1, however, the effect varied across growth stages, showing a significant growth stage × substrate × genotype interaction (Table 2A).

For example, at the end of week 3 in cycle 1, plantlets grown in single KlasmannTS3 had more internodes than those grown in K_25_P_75_, regardless of genotype (Table 5A). By week 4, plantlets of IBA70520 and IBA980505 had similar internode numbers in both substrates, while IBA961089A plantlets still had more internodes in single KlasmannTS3 compared to K_25_P_75_. Plantlets grown in single local peat or single vermiculite had similar internode numbers by the end of week 3, but by week 4, IBA961089A plantlets had more internodes in single local peat than in single vermiculite.

**Table 5:** Average number of internode (N°) per plantlet of cassava genotypes grown in different substrates across production cycles in the SAH laboratory.

Cycle	Growth stage	Genotype	Substrate					
			K_25_P_75_	KlasmannTS3	Local	V_10_P_90_	V_25_P_75_	Vermiculite
**1 (A)**	**Week 2**	**Overall**	1.2^b^	1.7^a^	0.9^bc^	0.6^c^	0.7^c^	0.6^c^
	**Week 3**	**IBA70520**	3.2^b^	3.7^a^	2.9^b^	2.0^c^	2.4^c^	2.1^c^
		**A961089A**	3.4^b^	4.7^a^	2.5^c^	1.9^d^	2.2^cd^	1.9^d^
		**IBA980505**	2.8^bc^	4.7^a^	2.5^c^	2.4^c^	3.0^b^	1.7^d^
	**Week 4**	**IBA70520**	4.0^a^	4.5^a^	3.2^b^	2.9^b^	3.2^b^	3.0^b^
		**A961089A**	5.0^b^	6.7^a^	4.1^c^	3.1^d^	3.1^d^	3.1^d^
		**IBA980505**	4.8^a^	5.1^a^	2.9^b^	2.9^b^	3.4^b^	3.1^b^
**2 (B)**	**Overall**	**IBA70520**	2.9^a^	3.2^a^	2.0^b^	1.9^bc^	2.3^b^	1.6^c^
		**A961089A**	3.9^b^	5.6^a^	2.3^d^	2.8^c^	2.9^c^	2.1^d^
		**IBA980505**	3.0^b^	4.3^a^	1.8^d^	1.9^d^	2.5^c^	1.8^d^
**3 (C)**	**Overall**	**IBA70520**	3.0^b^	3.5^a^	2.2^c^	2.1^c^	2.4^c^	1.5^d^
		**A961089A**	4.0^b^	5.4^a^	2.3^d^	3.0^c^	3.0^c^	2.0^d^
		**IBA980505**	3.1^b^	3.8^a^	1.6^d^	2.1^d^	2.6^c^	1.9^d^

Means with different letters in each row are significantly different (α < 5%) using the HSDT.

In cycle 3, all genotypes had more internodes in single KlasmannTS3 than in K_25_P_75_ throughout the growth period (Table 5C). In cycle 2, IBA961089A and IBA980505 plantlets had more internodes in single KlasmannTS3 compared to K_25_P_75_, while IBA70520 plantlets had similar internode numbers in both substrates, with these contrasts remaining consistent over time (Table 5B). Additionally, in cycles 2 and 3, IBA70520 plantlets grown in single local peat had more internodes than those grown in single vermiculite at all growth stages.

Overall, plantlets grown in single KlasmannTS3 developed more internodes than those grown in other single substrates or combinations of vermiculite and local peat, regardless of the cycle and growth stage. In cycles 2 and 3, plantlets grown in combinations of local peat and vermiculite (V_25_P_75_ and V_10_P_90_) generally had more internodes compared to those grown in single local peat or single vermiculite.

#### Leaf absorbance capacity

**Fig 3:**
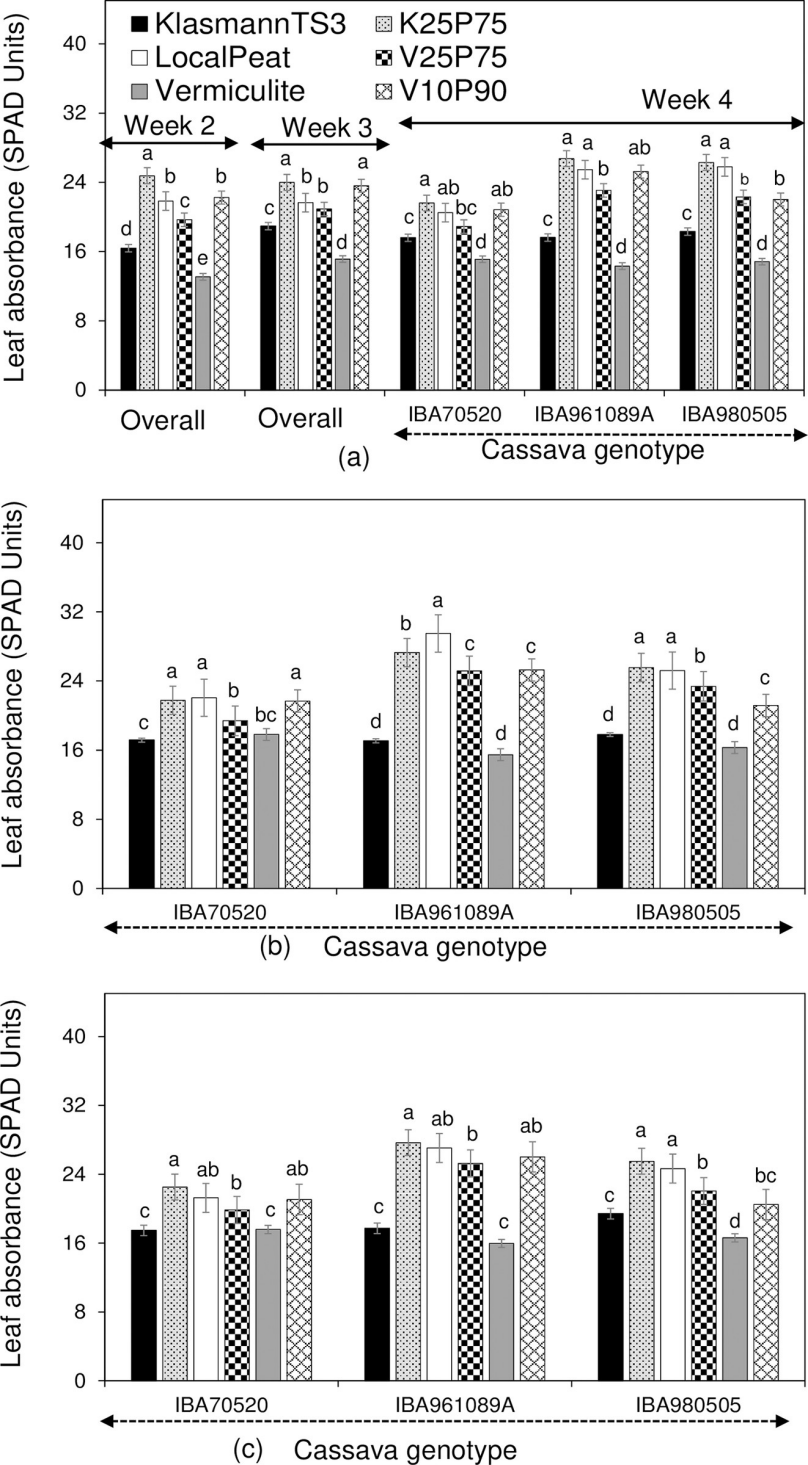
Leaf absorbance capacity of three cassava genotypes grown on six substrates across three production cycles in the SAH laboratory. (a): 1^st^ cycle, (b): 2^nd^ cycle, and (c): 3^rd^ cycle. Data at the end of weeks 2 and 3 in cycle 1 was not specified per genotype as substrate effect did not depend on the genotype. Data of cycles 2 and 3 were not specified per week as substrate or genotype effect did not depend on plantlet growth stage. Per genotype or per week, values of the histograms marked with different letters are significantly different (α < 5%) using the HSDT.

The absorbance capacity in cassava leaves differed significantly among the substrates and genotypes (Fig 3).

A significant interaction between substrate and cassava genotype was observed for leaf absorbance capacity at the end of production cycle. Despite these interactions, the differences among substrates remained consistent across all measurement times (Table 2D).

Across all cycles, genotypes, and growth stages, plantlets grown in single local peat consistently had higher chlorophyll content compared to those grown in the other single substrates. Plantlets grown in single KlasmannTS3 had a higher absorbance capacity than those grown in single vermiculite, especially in cycle 1 and for genotype IBA980505 in cycle 3. For the other two genotypes in cycle 3, and all genotypes in cycle 2, leaf absorbance capacity was similar between plantlets grown in single KlasmannTS3 and those grown in single vermiculite.

When local peat and KlasmannTS3 were combined (K_25_P_75_), the resulting plantlets generally had absorbance capacity at least equal to those grown in single local peat, with a notable increase at the early stage of cycle 2. Plantlets grown in K_25_P_75_ had higher leaf absorbance capacity than those grown in single KlasmannTS3, regardless of genotype and growth stage.

Combinations of local peat and vermiculite (V_10_P_90_ and V_25_P_75_) resulted in higher leaf absorbance capacity compared to single vermiculite. However, compared to single local peat, the leaf absorbance capacity of plantlets grown in these combinations varied with the proportion of local peat and the cassava genotype. For example, IBA980505 plantlets had lower leaf absorbance capacity in these combinations than in single local peat. For IBA961089A, plantlets in V_10_P_90_ or V_25_P_75_ had lower absorbance capacity in cycle 2, while in cycle 1, only V_25_P_75_ plantlets showed lower leaf absorbance capacity. For IBA70520, only in cycle 2 did V_25_P_75_ plantlets show lower leaf absorbance capacity compared to those in single local peat.

### Effect of substrate on plantlet rooting system

The number of the main roots in cassava plantlets differed significantly among the substrates, showing a significant interaction with the cassava genotype (Table 2F). Plantlets grown in single KlasmannTS3 and in the combination of KlasmannTS3 with local peat (K_25_P_75_) had the highest number of main roots in all production cycles (Table 6A).

**Table 6:** Average number (N°) and length (cm) of main roots at four weeks for cassava genotypes grown in different substrates across production cycles, in the SAH laboratory.

Parameter	Cycle	Genotype	K_25_P_75_	KlasmannTS3	Local Peat	V_10_P_90_	V_25_P_75_	Vermiculite
Root number (A)	1	Overall	7.1^a^	5.7^ab^	3.6^c^	4.3^bc^	4.1^bc^	3.5^c^
	2	Overall	6.7^a^	5.6^ab^	3.6^c^	4.3^bc^	4.1^bc^	3.4^c^
	3	Overall	6.0^a^	5.2^ab^	3.6^cd^	4.2^bc^	3.9^bc^	2.4^d^
Root length (B)	1	IBA70520	6.6c	6.1c	4.1^d^	6.0^c^	9.0^b^	10.7^a^
		IBA961089A	8.4^d^	9.3c	5.0^f^	7.0^e^	12.0^b^	13.3^a^
		IBA980505	9.4^b^	5.1^d^	4.1^e^	7.6^c^	10.8^a^	10.1^ab^
	2	IBA70520	6.6^c^	6.1^c^	4.1^d^	6.0^c^	8.8^b^	10.7^a^
		IBA961089A	8.1^d^	9.1^c^	5.0^f^	7.0^e^	11.8^b^	13.3^a^
		IBA980505	9.3^b^	5.1^d^	4.1^e^	7.8^c^	10.8^a^	10.0^b^
	3	IBA70520	6.6^c^	6.1^cd^	4.1^e^	6.0^d^	9.0^b^	11.0^a^
		IBA961089A	8.1^c^	8.6^c^	5.0^e^	7.0^d^	11.7^b^	13.2^a^
		IBA980505	9.0^b^	5.1^d^	4.1^e^	7.8^c^	10.6^a^	9.2^b^

Means with different letters in each row are significantly different (α < 5%) using the HSDT.

Plantlets grown in single local peat, single vermiculite, or combinations (V_25_P_75_ and V_10_P_90_) generally had similar numbers of main roots, except in cycle 3, where plantlets in single vermiculite had significantly fewer main roots compared to those in V_25_P_75_ and V_10_P_90_.

There was a significant interaction between substrate and genotype for main root length. Single vermiculite resulted in fewer main roots compared to single KlasmannTS3 but longer roots compared to other single substrates across all genotypes and cycles (Table 6B).

Plantlets in single KlasmannTS3 consistently had longer roots than those in single local peat. Combining KlasmannTS3 with local peat (K_25_P_75_) significantly increased main root length compared to single local peat, although roots were still shorter than in single KlasmannTS3, except for genotype IBA980505. Combining vermiculite and local peat at V_25_P_75_ resulted in shorter roots for IBA961089A and IBA70520 but longer roots for IBA980505 compared to single vermiculite. At V_10_P_90_, the combination led to shorter roots compared to single vermiculite but longer roots compared to single local peat, regardless of genotype. Combining local peat with vermiculite (V_25_P_75_) resulted in longer roots compared to combining with KlasmannTS3 (K_25_P_75_).

## Discussion

Results of this study indicated that, although local peat did not produce as many cassava cuttings as single KlasmannTS3, substituting 75% of KlasmannTS3 with local peat resulted in cutting production comparable to single KlasmannTS3 (Fig 1). Similarly, substituting 75% of vermiculite with local peat significantly increased cutting production compared to single vermiculite and local peat, producing nearly the same amount as single KlasmannTS3 (Fig 1). Higher cutting production in substrates involving KlasmannTS3 or its combinations resulted from faster plantlet growth, higher height, and more leaves and internodes, although these results varied among cassava genotypes, cycles, and growth stages (Tables 4 and 5, Fig 2).

These findings align with previous studies, which reported that KlasmannTS3 promotes faster plantlet growth in various crops, including cassava and yam (*Dioscorea* spp.), lettuce (*Lactuca Sativa L)*, marigold (*Tagetes L*.) [18, 37–39].

As observed in this study, [40] reported good performance of Amaranth (*Gomphrena globosa L*.) plantlets grown in a combination of garden soil and KlasmannTS3. However, our results contrast with [41], who found no difference in the height of tomato (*Solanum lycopersicum* L.) plantlets grown in single KlasmannTS3 and its combination with local materials. Plantlets grown in single local peat or combined with other substrates had the highest leaf absorbance capacity, attributed to the high nitrogen content in local peat (Fig 3, Table 1). However, despite similar leaf absorbance capacity, plantlet growth and cutting production were lower in single local peat compared to its combination with KlasmannTS3 (Figs 1 and 2). This suggests that plantlet performance is influenced by multiple substrate characteristics rather than a single physical or chemical property. For example, KlasmannTS3 and vermiculite had similar pH levels, but KlasmannTS3 outperformed vermiculite in plantlet growth and cutting production (Table 1, Fig 1). Additionally, although vermiculite had the highest exchangeable K and Mg content, it produced fewer cuttings than KlasmannTS3, indicating that external nutrients from weekly nutritive solutions might be the primary nutrient source for plantlets [28, 29, 42, 43].

Moreover, substrates may contain additional nutrients or possess characteristics not analyzed in this study. Literature suggests that commercial substrates often contain nutrients or hormones that promote plant growth [44, 45]. Additionally, [36] noted that these substrates provide a beneficial water-air balance, enhancing nutrient transport, absorption, and root system growth.

Plantlets grown in single KlasmannTS3 and its combination with local peat had the highest survival rates, likely due to the balanced texture of these substrates, which promote root adhesion and aeration (Table 3). This result aligns with those reported for cassava (*Manihot esculenta*) and pineapple (*Ananas comosus*) by [31, 34]. In contrast, the light texture of vermiculite and the heavy texture of local peat resulted in lower survival rates. Combining local peat with vermiculite improved plantlet survival rates by providing an intermediate texture, confirming the detrimental effects of extreme substrate textures on plantlet survival [38, 43–46].

Combining local peat and vermiculite increased plantlet survival compared to using either substrate alone, likely due to their intermediate texture (Table 3). These combinations are anticipated to have textures that fall between the extremes of vermiculite’s light texture and local peat’s heavy texture.

The study’s findings suggest that up to 75% of the imported KlasmannTS3 can be replaced by local peat without compromising cutting production, improving the cost-efficiency and availability of SAH laboratory.

KlasmannTS3 can be used in combination at slightly lower rates without affecting plantlet height or leaf development, although further reduction may decrease internode numbers (Table 5, Fig 2). The combination of local peat and vermiculite at 25% and 75%, respectively, produced cutting numbers similar to single KlasmannTS3, suggesting it as an alternative substrate. However, reducing the vermiculite rate in the combination below 25% compromised cutting production.

The multiplication rate of cuttings in this study was generally low, with less than twice the number of plantlets obtained in subsequent cycles (Fig 1), regardless of the substrate and cassava genotype. In some cases, the number of cuttings obtained was even lower than the number of plantlets that grew in the cycle. The multiplication rate of the clonal material using rapid multiplication technologies varies in the literature. For example, from 100 boxes of 25 plantlets each, [27] produced 1600 boxes within two months, corresponding to about eight cuttings obtained from a single cassava cutting in four weeks (ratio 1:8). From a single botanical yam seed, [33] obtained 310 plants in 161 days, corresponding to about fifty plants from a single yam seed within four weeks (ratio 1:50). These variations in multiplication rates can be attributed to various factors, including the crop, substrates, lighting, and temperature.

In this study, the cutting multiplication rate and all the plantlet growth parameters depended on the cassava genotype (Figs 1 and 3, and Table 5). This variability may explain why different multiplication rates are reported in the literature for the same crop, as different genotypes likely grow at different rates and produce varying numbers of leaves and internodes. Additionally, there was significant interaction between the cassava genotype and substrate regarding plantlet growth parameters (Table 2). Although single KlasmannTS3, its combination with local peat, and that of vermiculite with local peat generally performed better than other substrates, their performance varied among the cassava genotypes, and growth stage.

Plantlets grown in single vermiculite had the densest root system, longest main roots and the largest number of secondary roots (Table 6), but their survival in the field might be low due to the vermiculite’s light texture. Combining vermiculite with local peat produced improved root length, combining local peat with KlasmannTS3 increased the number of main roots. These results suggest that a combination of all the three substrates can enhance the rooting system without compromising cutting production.

We concluded that up to 75% of KlasmannTS3 or vermiculite can be substituted by local peat, without compromising cutting production like that of single KlasmannTS3. Based on the positive effect of vermiculite on the rooting system of plantlets, either when used alone or combined with local peat, we suggest that it contributes significantly to root development. Additionally, the higher cutting number that resulted from combining local peat with either KlasmannTS3 or vermiculite, indicates that these combinations are beneficial. Therefore, we argued that a combination of all three substrates should enhance the rooting system of the plantlets, while maintaining or improving cutting production.

## Supporting information

S1 Data (XLSX)

S2 Data (XLSX)

S3 Data (XLSX)

S4 Data (XLSX)

S5 Data (XLSX)

S6 Data (XLSX)

S7 Data (XLSX)

## References

[1] Feleke S, Manyong V, Abdoulaye T, Alene AD. Assessing the impacts of cassava technology on poverty reduction in Africa. Studies in Agricultural Economics. 2016;118(2):101–11. 10.7896/j.1612.

[2] Prudencio YC, Al-Hassan R. The food security stabilization roles of cassava in Africa. Food Policy. 1994;19(1):57–64. 10.1016/0306-9192(94)90008-6.

[3] Spencer DSC, Ezedinma C. Cassava cultivation in sub-Saharan Africa Chuma Ezedinma, UNIDO, Nigeria. Achieving Sustainable Cultivation cassava. 2017;1(October):141–157. Burleigh Dodds Science Publishing.

[4] Ramirez J, Bellotti AC, Jarvis A, Alvarez E. Adaptation of Cassava to Changing Climates. Crop Adaption To Climate Change 2011;411–25.

[5] FAOSTAT. Crop production quantity. Rome:FAOSTAT. 2020. https://www.fao.org/faostat/en/#data/QCL

[6] Mahungu N.M.; Bakelana Z.T; Kombo G.R.A; Bidiaka S.M. Origine, Domestication et Introduction du manioc en RD Congo. In Le Manioc en RDCongo. 2022. 1–13.

[7] Sikirou M, Musungayi E, Binzunga M, Tshiamala T, Tata-Hangy W, Miafuntila PA, et al. Impact of double invasion of Cassava Brown Streak and Root Necrosis Diseases on cassava production in the Democratic Republic of Congo. In: Plant and Animal Genome XXIX Conference (January 8–12, 2022). 2022.

[8] Bentley J, Nitturkar H, Friedmann M, Thiele G. BASICS Phase I—Final Report. 2020. https://hdl.handle.net/10568/110984

[9] Rwegasira GM, Rey CM. Response of selected cassava varieties to the incidence and severity of cassava brown streak disease in Tanzania. Journal of Agricultural Science. 2012;4(7):237. 10.5539/jas.v4n7p237

[10] Otoo JA. Principles of rapid multiplication. Vol. 51, IITA Research Guide 51. Ibadan, Nigeria; 1996. https://betuco.be/manioc/Rapid multiplication of cassava IITA

[11] Elegba W, Gruissem W, Vanderschuren H. Screening for resistance in farmer-preferred cassava cultivars from Ghana to a mixed infection of cbsv and ucbsv. Plants. 2020;9(8):1026. doi: 10.3390/plants9081026 32823622 PMC7465500

[12] Patil BL, Fauquet CM. Cassava mosaic geminiviruses: actual knowledge and perspectives. Molecular Plant Pathology. 2009;10(5):685–701. doi: 10.1111/j.1364-3703.2009.00559.x 19694957 PMC6640248

[13] Wosula EN, Chen W, Fei Z, Legg JP. Unravelling the genetic diversity among cassava bemisia tabaci whiteflies using NextRAD sequencing. Genome Biology and Evolution. 2017;9(11):2958–73. doi: 10.1093/gbe/evx219 29096025 PMC5714214

[14] Chen W, Wosula EN, Hasegawa DK, Casinga C, Shirima RR, Fiaboe KKM, et al. Genome of the African cassava whitefly Bemisia tabaci and distribution and genetic diversity of cassava-colonizing whiteflies in Africa. Insect Biochemistry and Molecular Biology. 2019;110(May):112–20. doi: 10.1016/j.ibmb.2019.05.003 31102651

[15] Legg JP, Diebiru-Ojo E, Eagle D, Friedmann M, Kanju E, Kapinga R, et al. Commercially sustainable cassava seed systems in Africa. Root, Tuber Banana and Food Syst Innov. 2022;453–82.

[16] Ceballos H, Rojanaridpiched C, Phumichai C. Excellence in cassava breeding: perspectives for the future. Crop Breeding, Genetics Genomics. 2020;2:1–31. 10.20900/cbgg20200008

[17] Pelemo O, Koeyer D De, Matsumoto R, Agre P, Asiedu R, Asfaw A. Semi-Autotrophic Hydroponics: A robust technique for accelerated basic seed yam production. 2019. IITA-Space. 10.13140/RG.2.2.26336.74249

[18] Thiele G, Friedmann M, Campos H, Polar V. Root, tuber and banana food system innovations: Value creation for inclusive outcomes. Springer Nature. 2022. 561.

[19] Klasmann-Deilmann. Growing media for commercial horticulture. Easy Growing. 2019;27. https://klasmann-deilmann.com/wp-content/uploads/8982_KD_Aktualisierung_Easy_Growing.

[20] Adesanya TA, Adetoro NA, Olasupo TK, Aina OO, Iluebbey P, Agbona A, et al. SP10-6 Influence of Growth Nutrient and Rooting Hormone on Survival and Growth of Semi- Autotrophic Hydroponics (SAH TM) Cassava Plantlets. 2016;10. SP106.https://biblio1.iita.org/bitstream/handle/20.500.12478/5233/U18ProcOgwucheInfluenceNothomNodev.pdf?sequence=1

[21] Kajibwami A, Kintche K, Okafor C, Nabahungu L, Casinga C. SAH technology to produce cassava plantlets, a fascinating and learning process. 2018. International Institute of Tropical Agriculture.

[22] Pelemo O, Benjamin G, Adejumobi I, Olusola T, Odom-Kolombia O, Adeosun T AA. Semi-Autotrophic Hydroponics: A potential seed system technology for reduced breeding cycle and rapid quality seed delivery. 2019.

[23] Olagunju YO, Aduloju AO, Akin-Idowu PE, Esuola CO. Acclimatization of tissue culture pineapple plantlet using Semi-autotrophic hydroponics technique in comparison with other conventional substrates. Journal of Experimental Agriculture International. 2021;43(April):61–7. 10.9734/jeai/2021/v43i1130757.

[24] Klougart A. Substrates and nutrient flow. In: International Symposium on Substrates in Horticulture other than Soils In Situ 150. 1983. 297–314.

[25] Maucieri C, Nicoletto C, van Os E, Anseeuw D, Van Havermaet R, Junge R. Hydroponic technologies. Aquaponics Food Production Systems. 2019. 77–110. 10.1007/978-3-030-15943-6_4.

[26] Goddek S, Joyce A, Kotzen B, Burnell GM. Correction to: Aquaponics food production systems: Combined Aquaculture and Hydroponic Production Technologies for the Future. Springer Nature. 2019. 10.1007/978-3-030-15943-6_25.

[27] Awad YM, Lee SE, Ahmed MBM, Vu NT, Farooq M, Kim IS, et al. Biochar, a potential hydroponic growth substrate, enhances the nutritional status and growth of leafy vegetables. Journal of Cleaner Production. 2017. 156:581–8. 10.1016/j.jclepro.2017.04.070.

[28] Binzunga M M, Kintche K, Mouritala S, Kajibwami A NA and ABN. Performances of plantlets from selected cassava (*Manihot esculenta* Crantz) genotypes under Semi—Autotrophic Hydroponics (SAH) using different substrates. Journal of Agriculture, Science and Technology. 2023. 22(6):66–89. 10.4314/jagst.v23i6.5.

[29] Parkinson JA, Allen SE. A wet oxidation procedure suitable for the determination of nitrogen and mineral nutrients in biological material. Communications in Soil Science and Plant Analysis. 1975;6(1):1–11. 10.1080/00103627509366539.

[30] Thomas GW. Soil pH and soil acidity. Methods soil Analysis part 3 Chemical methods. 1996;5:475–90.

[31] Simard RR, Zizka J. Evaluating plant available potassium with strontium citrate. Communications in Soil Science and Plant Analysis. 1994;25(9–10):1779–89. 10.1080/00103629409369152.

[32] Ross DS, Ketterings Q. Recommended methods for determining soil cation exchange capacity. Recommended soil testing procedures for the northeastern United States. 1995;493(101):62.

[33] Bray RH, Kurtz LT. Determination of total, organic, and available forms of phosphorus in soils. Soil Science. 1945;59(1):39–46.

[34] Süb A, Danner M, Obster C, Locherer M, Hank T, Richter K. Measuring leaf chlorophyll content with the Konica minolta SPAD-502Plus. Field Guides Technical Report, GFZ Data Services. 2015;1–13. 10.2312/enmap.2015.010.

[35] McCullagh P, Nelder JA. Generalized linear models. Routledge. Springer; 2019.

[36] Institute SAS. The SAS System for Windows. Cary, NC, USA, SAS Institute Inc.; 2019.

[37] Ogwuche TO, Adesanya TA, Diebiru-Ojo EM, Adetoro NA, Olasupo KT, Kumar P, et al. Influence of growth nutrient and rooting hormone on survival and growth of Semi- autotrophic hydroponics (SAH TM)cassava plantlets. SP10–6. 2016. https://biblio1.iita.org/bitstream/handle/20.500.12478/5233/U18ProcOgwucheInfluenceNothomNodev.pdf?sequence=1

[38] Balalic I. Effects of production method and substrate on yield and quality of lettuce (*Lactuca sativa* L.). AGRIS—International System for Agricultural Science and Technology. 2004;105–8.

[39] Maślanka M, Magdziarz R. The influence of substrate type and chlormequat on the growth and flowering of marigold (*Tagetes* L.). Folia Horticulturae. 2017;29(2):189–98. 10.1515/fhort-2017-0018

[40] Zeljković S, Ećim T, Davidović Gidas J, Mladenović E. Effects of different substrates on growth and development of Globe amaranth (*Gomphrena globosa* L.). Agro-Knowledge Journal. 2021;22(4):107–16. 10.7251/agren2104107z42.

[41] Manios I V, Syminis IC, Kritsotakis KI. Substrates for growth of tomato seedlings. Georg Erevna. 1987;(11):(2).

[42] Li Y, He N, Hou J, Xu L, Liu C, Zhang J, et al. Factors influencing leaf chlorophyll content in natural forests at the biome scale. Frontiers (Boulder). 2018;6(JUN):1–10. 10.3389/fevo.2018.00064.

[43] Croft H, Chen JM, Luo X, Bartlett P, Chen B, Staebler RM. Leaf chlorophyll content as a proxy for leaf photosynthetic capacity. Global Change Biology. 2017;23(9):3513–24. doi: 10.1111/gcb.13599 27976452

[44] Khalaj MA, Azimi MH, Sayyad-amin P. The effect of different growth Media on cala Lily (*Zantedeschia* spp.). Journal of Ornamental Plants. 2022;12(4):279–86.

[45] Savvas D, Gruda NS. Application of soilless culture technologies in the modern greenhouse industry–A review. European Journal of Horticultural Science. ·. 2018;83(5):280–93. 10.17660/eJHS.2018/83.5.2.

[46] Khan S, Purohit A, Vadsaria N. Hydroponics: current and future state of the art in farming. Journal of Plant Nutrition. 2020;44(10):1515–38. 10.1080/01904167.2020.1860217.

[47] Jones JB. Hydroponics: Its history and use in plant nutrition studies. 1982;5(8):1003–30. 10.1080/01904168209363035.

[48] Santiago-Aviles JJ, Light G. Embedded controlled gardening: An academically based service course. In 2018 IEEE integrated STEM education conference (ISEC) 2018 Mar 10. 2018;149–53.

